# Overexpression of Rab1B and MMP9 predicts poor survival and good response to chemotherapy in patients with colorectal cancer

**DOI:** 10.18632/aging.101200

**Published:** 2017-03-18

**Authors:** Xian-Zi Yang, Shu-Zhong Cui, Li-Si Zeng, Tian-Tian Cheng, Xiao-Xing Li, Jun Chi, Ren Wang, X.F. Steven Zheng, Hui-Yun Wang

**Affiliations:** ^1^ State Key Laboratory of Oncology in South China, Sun Yat-Sen University Cancer Center, Guangzhou, Guangdong, 510060, China; ^2^ Collaborative Innovation Center for Cancer Medicine, Sun Yat-Sen University Cancer Center, Guangzhou, Guangdong, 510060, China; ^3^ Department of Abdominal Surgery, Affiliated Cancer Hospital & Institute of Guangzhou Medical University of Guangzhou Medical University, Guangzhou Medical University, Guangzhou, Guangdong, 510095, China; ^4^ Department of Endoscopy and Laser, Sun Yat-Sen University Cancer Center, Guangzhou, Guangdong, 510060, China; ^5^ Rutgers Cancer Institute of New Jersey, Department of Pharmacology, Robert Wood Johnson Medical School, Rutgers, The State University of New Jersey, New Brunswick, NJ 08901, USA

**Keywords:** colorectal cancer, Rab1B, MMP9, prognosis, adjuvant chemotherapy

## Abstract

Rab1B has recently been reported to be involved in human cancer, but the role of Rab1B in colorectal cancer (CRC) remains unclear. In this study, we investigated the expression of Rab1B and MMP9 in CRC by qRT-PCR, immunoblot and immunohistochemistry and analyzed the clinical significance. The results show that Rab1B and MMP9 are increased at both mRNA and protein levels in CRC cell lines and tissues, as measured by qRT-PCR and immunoblotting. The high protein expression of Rab1B and MMP9 in 179 CRC tissues is associated with deep tumor invasion, lymph-node metastasis and advanced TNM stage. Survival analysis indicates that patients with overexpression of Rab1B or MMP9 have significantly worse overall survival and progression-free survival, but better response to chemotherapy than those with low expression of proteins, and that Rab1B is an independent prognostic factor for CRC patients. Furthermore, when Rab1B and MMP9 are combined into a new risk model, it has a remarkably better prediction of prognosis than each protein alone. In conclusion, Rab1B and MMP9 are potential prognostic biomarkers and their combination significantly improves predictive power for survival and chemotherapy response in CRC patients.

## INTRODUCTION

Colorectal cancer (CRC) is the third most common cancer in men worldwide. Although the 5-year survival rate of CRC patients is more than 60%, it is still the second leading cause of cancer-related death in the developed countries [1, 2]. The primary reason for the high mortality of CRC is due to its high recurrence and metastasis in approximately half of all the patients, which are extremely difficult to cure [2, 3]. The standard therapy for CRC patients with stage III and high-risk stage II is resection plus postoperative adjuvant chemotherapy [4]. However, a subpopulation of the patients does not benefit from adjuvant chemotherapy, while suffering long-term toxicity [5]. At present, the commonly high-risk features, such as poor differentia-tion, T4 tumors and margin involvement, cannot precisely distinguish patients with high-risk from low-risk for disease recurrence and metastasis [6]. Because tumor behaviors are determined by oncogenic alterations in molecular and cellular processes, such aberrant events can be explored as predictive biomarkers for the progression and metastasis of human cancer. Moreover, a better understanding of the molecular mechanisms under-lying the progression and metastatic process of CRC can lead to new cancer drug target [7]. There is a pressing need for identifying key molecular markers for prediction of recurrence, metastasis and chemotherapeutic outcome to improve the survival of patients with CRC.

Rab1A and Rab1B share highly homologous sequence and function in membrane trafficking between endoplasmic reticulum to Golgi apparatus [8]. They have also been reported to be involved in the progression and metastasis of human cancer by regulating different cell signaling pathways [9]. In the previous studies, we demonstrated that Rab1A is overexpressed and associated with tumor prognosis in patients with CRC and hepatocellular carcinoma (HCC), promoting tumor growth and metastasis through activating mTORC1 signaling [10, 11]. It was reported that Rab1A enhances migration of breast cancer cells through promotion of ITGB1 recycling to the plasma membrane [12]. RablA overexpression was also reported in human lung cancer, which is correlated with tumor volume and stage, but the tumorigenic function of Rab1A is not dependent on mTOR or MAPK signaling [13]. These results suggest that in different types of tumor, Rab1A promotes cell migration and invasion through distinct mechanisms. Rab1B, the second Rab1 member, was also found to be increased in cervical cancer [14] and HCC [15], but another study showed that Rab1B protein was down-regulated and inhibited tumor proliferation and migration via regulating TGF-β/Smad pathway in triple-negative breast cancer (TNBC) [16], indicating that Rab1B may have different roles in different cancer types. Recently, Zhai et al reported that Rab1B mRNA was increased in a small sample size (23 cases) of CRC [17]. However, the clinical significance of Rab1B expression in CRC patients has not been evaluated.

Matrix metalloproteinases (MMPs) play an important role in degradation of extracellular matrix (ECM) and basement membranes (BM). In CRC, previous studies indicate that the expression of MMP9 is associated with metastasis [18-20] and poor prognosis [21-23]. Interestingly, a recent study showed that Rab1A knockdown decreases MMP9 expression and inhibites MMP9-mediated invasiveness of human oral squamous cell cancer cells, suggesting that Rab1A regulates MMP9-mediated invasiveness [24]. Given the high homology between Rab1A and Rab1B, it would be interesting to determine whether Rab1B protein promotes invasiveness and metastasis by upregulating the expression of MMP9 in CRC.

In this study, we investigated the expression of Rab1B and MMP9 proteins and their relationship in CRC tissues and cell lines. We further analyzed the correlation between Rab1B and MMP9 expressions and clinicopathological parameters as well as prognosis in CRC patients. Finally, we evaluated the predictive value of Rab1B and/or MMP9 protein expressions in CRC patients who underwent chemotherapy treatment.

## RESULTS

### The expression of Rab1B and MMP9 is up-regulated in CRC cell lines

**Figure 1:**
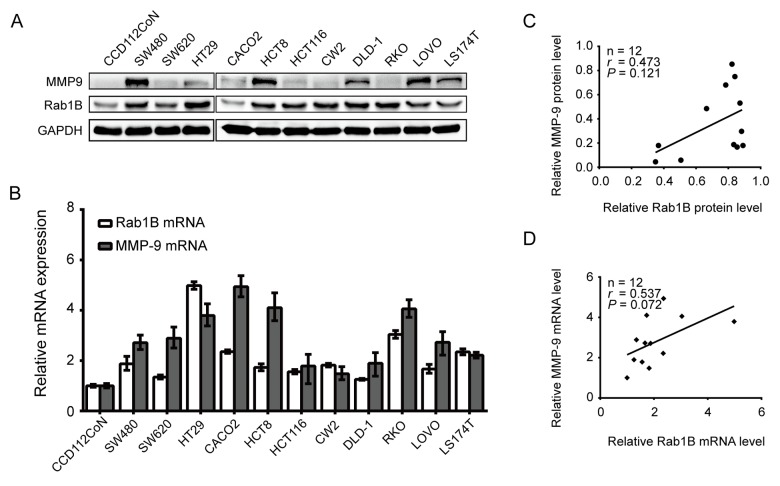
Rab1B and MMP9 are overexpressed in CRC cell lines (**A**) The expression of Rab1B and MMP9 proteins in a panel of CRC cell lines and an immortalized colon cell line is determined by immunoblot. GAPDH is used as a loading control. (**B**) Relative expression of Rab1B and MMP9 mRNA (normalized to GAPDH) in the same set of cell lines as in (**A**) is examined by real-time quantitative PCR. (**C**) The correlation between Rab1B and MMP9 proteins (normalized to GAPDH) in the CRC cell lines was determined by Spearman correlation assay. (**D**) Spearman correlation analysis is used to analyze the correlation between Rab1B and MMP9 mRNAs in CRC cell lines.

To determine the expression of Rab1B and MMP9 in CRC, we first performed immunoblot and RT-PCR in 11 CRC cell lines and one human normal colon cell line CCD112CoN. The result showed that the expression levels of Rab1B and MMP9 proteins were significantly increased in 81.8 % (9/11) and 63.6% (7/11) of CRC cell lines, respectively, compared with those in normal cell line (Fig. 1A). As expected, relative expressions of Rab1B and MMP9 mRNA in CRC cell lines were also higher than those in CCD112CoN cells (Fig. 1B). Furthermore, we found that there is a positive correlation trend between Rab1B and MMP9 expression in both mRNA and protein (for mRNA: *r* = 0.473, *P*=0.121; for protein: *r* = 0.537, *P* = 0.072, Fig. 1C and 1D), although the correlations were not statistically significant. These results indicate that both Rab1B and MMP9 are up-regulated in CRC cells and may have a positive correlation.

### The protein expression of Rab1B and MMP9 is increased and has a positive correlation with each other in CRC tissues

**Figure 2:**
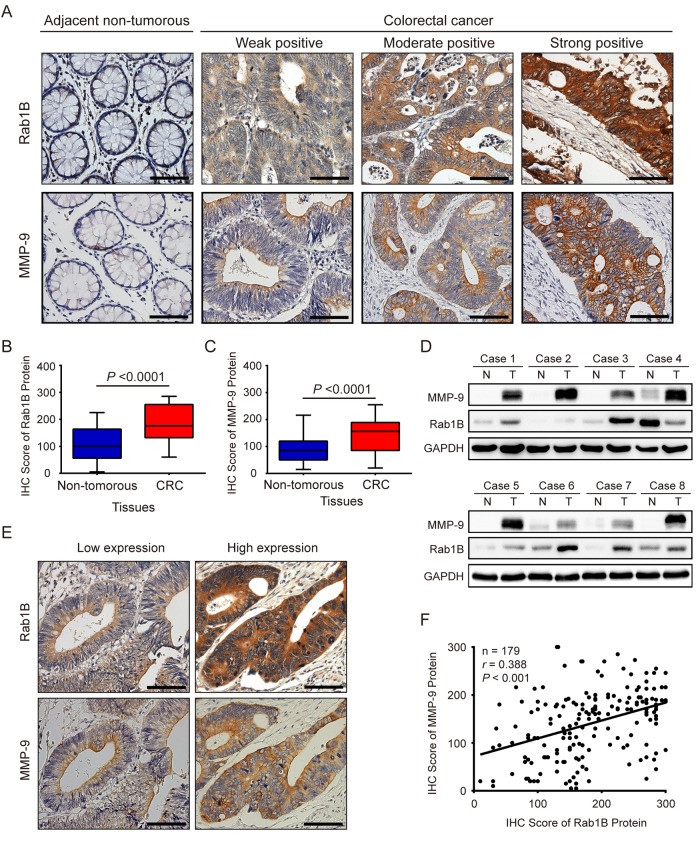
Rab1B and MMP9 expressions are significantly increased in colorectal cancer tissues (**A**) **Shown are r**epresentative immunohistochemistry (IHC) staining of Rab1B and MMP9 in CRC and adjacent non-tumor tissues. Scale bars represent 50 μm. (**B**) Comparison of Rab1B protein expressions between CRC tissues and matched adjacent non-tumorous tissues. (**C**) Comparison of MMP9 protein expressions between CRC tissues and matched adjacent non-tumorous tissues. (**D**) The expression levels of Rab1B and MMP9 proteins in eight pairs of CRC tissues (T) and adjacent non-tumorous tissues (N) are analyzed by immunoblot. (**E**) Concordance of Rab1B and MMP9 expressions in CRC. Consecutive CRC sections were analyzed for the expression of Rab1B and MMP9 by IHC. Shown are two representative cases. Scale bars represent 50 μm. (**F)** The correlation between Rab1B and MMP9 protein expressions in CRC tissues as evaluated by Spearman correlation analysis.

To further investigate the expression and clinical significance of Rab1B and MMP9 protein in CRC, we collected 179 pairs of cancer and corresponding adjacent non-tumor tissues from CRC patients. Their demographic and clinicopathological data are shown in Table 1. The protein expression of Rab1B and MMP9 in the paired CRC and non-tumor tissues was examined by immunohistochemistry (IHC). Both Rab1B and MMP9 proteins are mainly distributed in the cytoplasm and membrane of CRC cells (Fig. 2A) with 81% (145/179) and 71.5% (128/179) of CRC samples displaying higher expression of Rab1B and MMP9 proteins than the matched adjacent non-tumorous tissues, respectively (*P* <0.0001, Fig. 2B and 2C). To verify the IHC results, we performed immunoblot analysis on another 8 paired CRC and non-tumorous tissues. As expected, high expression of Rab1B and MMP9 was found in 75% (6/8) and 100% CRC tissues (Fig. 2D), respectively, which is consistent with the results of IHC.

**Table 1:** Clinical characteristics of patients with colorectal cancer

Characteristics	N (%)
Gender	
Male	95 (53.1%)
Female	84 (46.9%)
Age (years)	
<65	117 (65.4%)
≥ 65	62 (34.6%)
Pathological grade	
I-II	147 (82.1%)
III-IV	32 (17.9%)
Tumor location	
Colon	94 (52.5%)
Rectum	85 (47.5%)
Tumor size	
< 5 cm	97 (54.2%)
≥ 5 cm	80 (44.7%)
Tumor depth	
Shallow	21 (11.7%)
Deep	68 (38.0%)
N stage	
N0	99 (55.3%)
N1-2	80 (44.7%)
TNM stage	
I	17 (9.5%)
II	81 (45.3%)
III	81 (45.3%)
Intraoperative chemotherapy	
No	126 (70.4%)
Yes	53 (29.6%)
Adjuvant chemotherapy	
No	109 (60.9%)
Yes	70 (39.1%)
Preoperative CEA (ng/ml)	
0-5	127 (70.9%)
> 5	52 (29.1%)
Preoperative CA199 (ng/ml)	
0-35	154 (86.0%)
> 35	25 (14.0%)

Shallow: the depth of tumor invasion within mucosa and muscularis; Deep: the depth of tumor invasion beyond serosa; TNM, tumor node metastasis; N, lymph node; CEA, Carcinoembryonic antigen; CA199, carbohydrate antigen 199

Furthermore, we analyzed the relationship between Rab1B and MMP9 expressions. In the same cohort of CRC tissue samples, 71.1 % (64/90) samples with MMP9 overexpression had high expression of Rab1B protein, while 69.7 % (62/89) samples with MMP9 down-regulation also had low expression of Rab1B (P < 0.001, Table 2 and Fig. 2E). Pearson's correlation analysis showed that there is a positive correlation between Rab1B and MMP9 protein expressions in 179 CRC samples (Fig. 2F, *r* =0.388, *P*< 0.001), which is similar to that in CRC cell lines. These data show that Rab1B and MMP9 proteins are significantly co-overexpressed in CRC tissues.

**Table 2:** The relationships of Rab1B and MMP9 expressions with clinicopathological characteristics in patients with colorectal cancer

Clinicopathological characteristics	Rab1B Expression		*P* value	MMP9 Expression		*P* value
	Low	High		Low	High	
Gender						
Male	45 (47.4%)	50 (52.6%)	0.503	48 (50.5%)	47 (49.5%)	0.698
Female	44 (52.4%)	40 (47.6%)		40 (47.6%)	44 (52.4%)	
Age (years)						
<65	55 (47.0%)	62 (53.0%)	0.319	56 (47.9%)	61 (52.1%)	0.633
≥ 65	34 (54.8%)	28 (45.2%)		32 (51.6%)	30 (48.4%)	
Tumor location						
Colon	42 (44.7%)	52 (55.3%)	0.156	45 (47.9%)	49 (52.1%)	0.717
Rectum	47 (55.3%)	38 (44.7%)		43 (50.6%)	42 (49.4%)	
Tumor size						
< 5 cm	52 (52.5%)	47 (47.5%)	0.404	53 (53.5%)	46 (46.5%)	0.193
≥ 5 cm	37 (46.3%)	43 (53.8%)		35 (43.8%)	45 (56.2%)	
Pathological grade						
I-II	73 (49.7%)	74 (50.3%)	0.972	74 (50.3%)	73 (49.7%)	0.499
III-IV	16 (50.0%)	16 (50.0%)		14 (43.8%)	18 (56.2%)	
Tumor depth						
Shallow	21 (91.3%)	2 (8.7%)	<0.001	18 (78.3%)	5 (21.7%)	0.003
Deep	68 (43.6%)	88 (56.4%)		70 (44.9%)	86 (55.1%)	
N stage						
N0	59 (59.6%)	40 (40.4%)	0.003	67 (67.7%)	32 (32.3%)	0.000
N1-2	30 (37.5%)	50 (62.5%)		21 (26.2%)	59 (73.8%)	
TNM stage						
I	15 (88.2%)	2 (11.8%)	0.001	16 (94.1%)	1 (5.9%)	<0.001
II	43 (53.1%)	38 (46.9%)		50 (61.7%)	31 (38.3%)	
III	31 (38.3%)	50 (61.7%)		22 (27.2%)	59 (72.8%)	
Intraoperative chemotherapy						
No	67 (53.2%)	59 (46.8%)	0.154	65 (51.6%)	61 (48.4%)	0.317
Yes	22 (41.5%)	31 (58.5%)		23 (43.4%)	30 (56.6%)	
Adjuvant chemotherapy						
No	57 (52.3%)	52 (47.7%)	0.390	56 (51.4%)	53 (48.6%)	0.460
Yes	32 (45.7%)	38 (54.3%)		32 (45.7%)	38 (54.3%)	
Preoperative CEA (ng/ml)						
0-5	62 (48.8%)	65 (51.2%)	0.706	62 (48.8%)	65 (51.2%)	0.886
> 5	27 (51.9%)	25 (48.1%)		26 (50.0%)	26 (50.0%)	
Preoperative CA199 (ng/ml)						
0-35	79 (51.3%)	75 (48.7%)	0.295	78 (50.6%)	76 (49.4%)	0.323
> 35	10 (40.0%)	15 (60.0%)		10 (40.0%)	15 (60.0%)	
Rab1B expression						
Low	-	-		62 (69.7%)	27 (30.3%)	<0.001
High	-	-	26 (28.9%)	64 (71.1%)	
MMP9 expression						
Low	62 (70.5%)	26 (29.5%)	<0.001	-	-	
High	27 (29.7%)	64 (70.3%)		-	-

### Rab1B and MMP9 overexpression is correlated with tumor progression and metastasis in CRC patients

To evaluate the clinical relevance of Rab1B and MMP9 protein expression in CRC patients, the median IHC scores of 180 and 156 were defined as the cutoff value for high- and low-expression of Rab1B and MMP9, respectively, which divided CRC patients into high- or low-expression groups. As showed in Table 2, high Rab1B and MMP9 protein expressions in CRC are significantly associated with deep invasion, lymph node metastasis, and advanced TNM stage, suggesting that both Rab1B and MMP9 are involved in the progression and metastasis of CRC.

### Rab1B and MMP9 protein expressions are associated with poor prognosis

**Figure 3:**
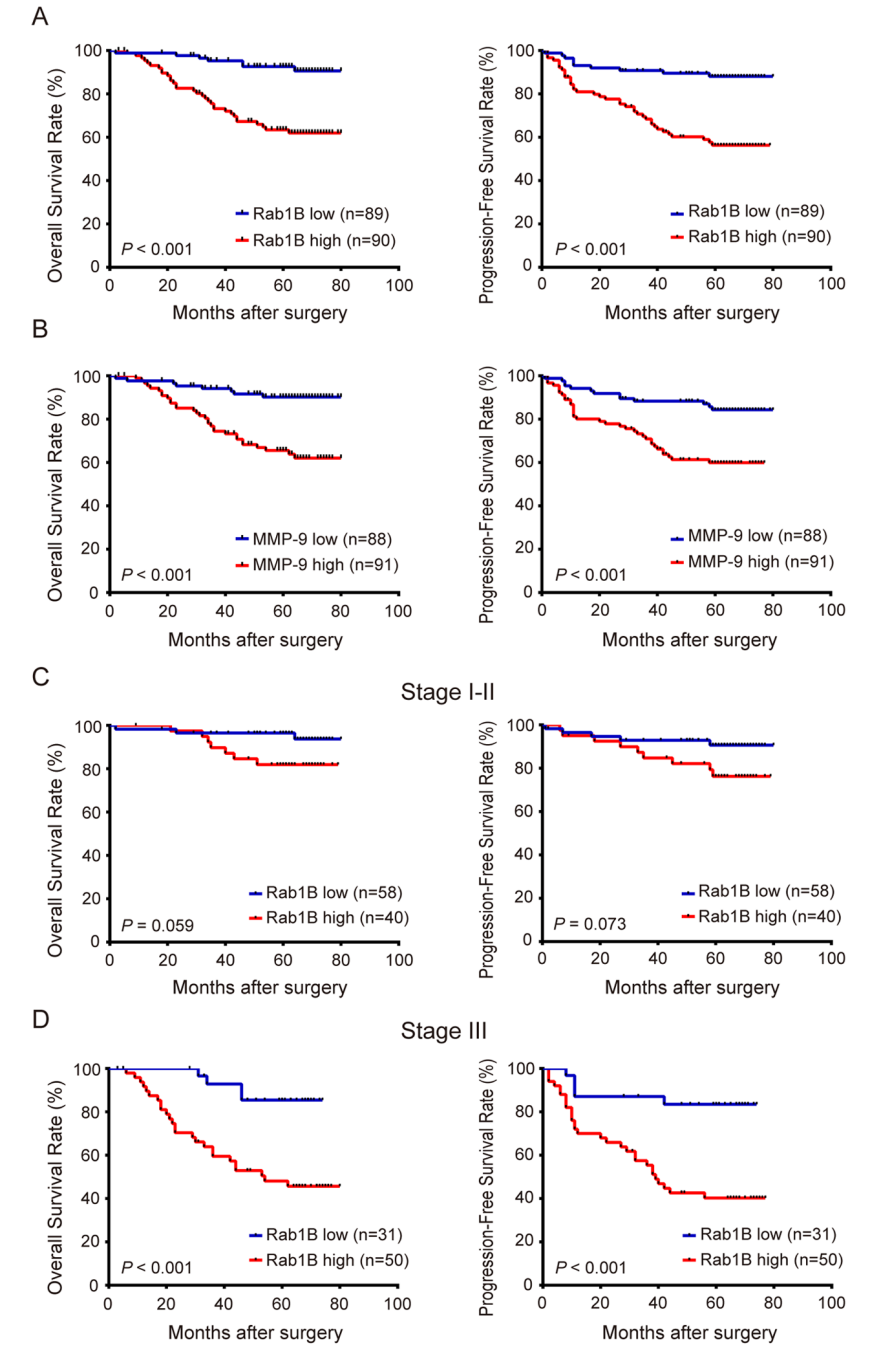
Overexpression ofRab1B and MMP9 proteins are associated with poor prognosis of CRC patients independent of clinical stage (**A**) The overall survival (OS) and progression-free survival (PFS) of CRC patients with high or low Rab1B expression. P value was calculated by Log-rank test. (**B**) OS and PFS of patients with high or low MMP9 expression. (**C**) OS and PFS of stage I-II CRC patients with high or low Rab1B expression. (**D**) OS and PFS of stage III CRC patients with high or low Rab1B expression.

To explore the prognostic value of Rab1B and MMP9 proteins in this disease, we analyzed overall survival (OS) and progression-free survival (PFS) of CRC patients with Rab1B and/or MMP9 expressions. Patients with Rab1B high-expression have significantly shorter 5-year OS rate (63.5 % vs. 92.6 %) and 5-year PFS rate (56.3 % vs. 88.1 %) than those with Rab1B low-expression (All *P* < 0.001, Fig. 3A). Similarly, patients with MMP9 high-expression have much poorer OS and PFS than those with MMP9 low-expression (Fig. 3B), which is consistent with the conclusion from a meta-analysis of 13 cohort studies on MMP9 expression and prognosis in CRC [25]. These results indicate that the increased Rab1B or MMP9 protein is significantly correlated with poor prognosis of CRC patients.

We next analyzed the effect of their expressions and clinicopathological parameters on patient survival by using univariate and multivariate Cox model. Univariate analysis indicates that TNM stage, adjuvant chemotherapy, Rab1B and MMP9 proteins are significant predictors for OS and PFS of CRC patients (All *P* < 0.05, Table 3), and Tumor depth is a marginally significant predictor for OS and PFS (*P* =0.065 and *P* =0.059, respectively, Table 3). Multivariate Cox regression analysis further demons-trates that Rab1B protein is an independent risk predictor for OS (HR: 3.605, 95% CI: 1.481-8.775, *P* = 0.005) and PFS (HR: 3.394, 95% CI: 1.579-7.297, *P* = 0.002) of CRC patients (Table 3), and MMP9 protein is a marginally significant independent unfavorable predictor for OS and PFS in CRC patients (Table 3). In addition, TNM stage and adjuvant chemotherapy also are independent prognostic factors for OS and PFS in CRC patients.

**Table 3:** Cox regression analysis of Rab1B, MMP9 and clinical characteristics associated with survival in patients with colorectal cancer

Variables	Univariate analysis		*P* value	Multivariate analysis		*P* value
	HR	95% CI		HR	95% CI	
**Overall Survival**						
Gender (Female vs. Male)	0.862	0.458-1.623	0.645			
Age (≥65y vs. <65y)	1.196	0.621-2.301	0.593			
Tumor location (Rectum vs. Colon)	0.778	0.413-1.465	0.436			
Tumor size (≥5cm vs. <5cm)	1.160	0.857-3.023	0.139			
Pathological grade (III-IV vs. I-II)	1.572	0.766-3.226	0.218			
Tumor depth (Deep vs. Shallow)	6.477	0.889-47.176	0.065	1.541	0.187-12.716	0.688
TNM stage (III vs. II vs. I)	3.971	2.028-7.778	<0.001	2.356	1.123-4.944	0.023
Intraoperative chemotherapy (Yes vs. No)	1.348	0.700-2.593	0.372			
Adjuvant chemotherapy (Yes vs. No)	0.375	0.172-0.815	0.013	0.372	0.168-0.824	0.015
Preoperative serum CEA (> 5 ng/ml vs. 0-5 ng/ml)	0.937	0.46-1.922	0.858			
Preoperative serum CA199 (> 35 ng/ml vs. 0-35 ng/ml)	1.120	0.469-2.674	0.798			
Rab1B expression (High vs. Low)	5.274	2.327-11.952	<0.001	3.605	1.481-8.775	0.005
MMP9 expression (High vs. Low)	4.403	2.022-9.586	<0.001	2.031	0.872-4.729	0.101
						
**Progression-Free Survival**						
Gender (Female vs. Male)	0.934	0.530-1.649	0.815			
Age (≥65y vs. <65y)	1.521	0.857-2.701	0.152			
Tumor location (Rectum vs. Colon)	0.739	0.416-1.312	0.302			
Tumor size (≥5cm vs. <5cm)	1.620	0.917-2.859	0.096			
Pathological grade(III-IV vs. I-II)	1.137	0.566-2.281	0.719			
Tumor depth (Deep vs. Shallow)	3.914	0.950-16.125	0.059	1.135	0.240-5.365	0.873
TNM stage (III vs. II vs. I)	3.083	1.762-5.394	<0.001	2.022	1.080-3.788	0.028
Intraoperative chemotherapy (Yes vs. No)	1.171	0.643-2.135	0.605			
Adjuvant chemotherapy (Yes vs. No)	0.428	0.218-0.839	0.013	0.415	0.207-0.831	0.014
Preoperative serum CEA (>5 ng/ml vs. 0-5 ng/ml)	1.110	0.596-2.069	0.742			
Preoperative serum CA199 (>35 ng/ml vs. 0-35 ng/ml)	1.272	0.595-2.718	0.535			
Rab1B expression (High vs. Low)	4.356	2.169-8.747	<0.001	3.394	1.579-7.297	0.002
MMP9 expression (High vs. Low)	3.031	1.602-5.735	0.001	1.608	0.793-3.259	0.188

Moreover, we found when patients were stratified by TNM stage, stage I-II patients with high level of Rab1B protein has marginally significantly poorer OS and PFS than those with low level (Fig. 3C), and stage III patients with high Rab1B level has significantly poorer survival than those with low level (Fig. 3D). This result reveals that Rab1B can predict survival of CRC patients independent of clinical stage. Therefore, Rab1B may provide additional prognostic information to the current clinical staging system.

Shallow: tumor invasion to mucosa and muscularis; Deep: tumor invasion to or beyond serosa; TNM, tumour node metastasis; N, lymph node; CEA, Carcinoembryonic antigen; CA199, carbohydrate antigen 199.

### Combination of Rab1B and MMP9 expression significantly improves predictive efficiency for the outcome of CRC patients

**Figure 4:**
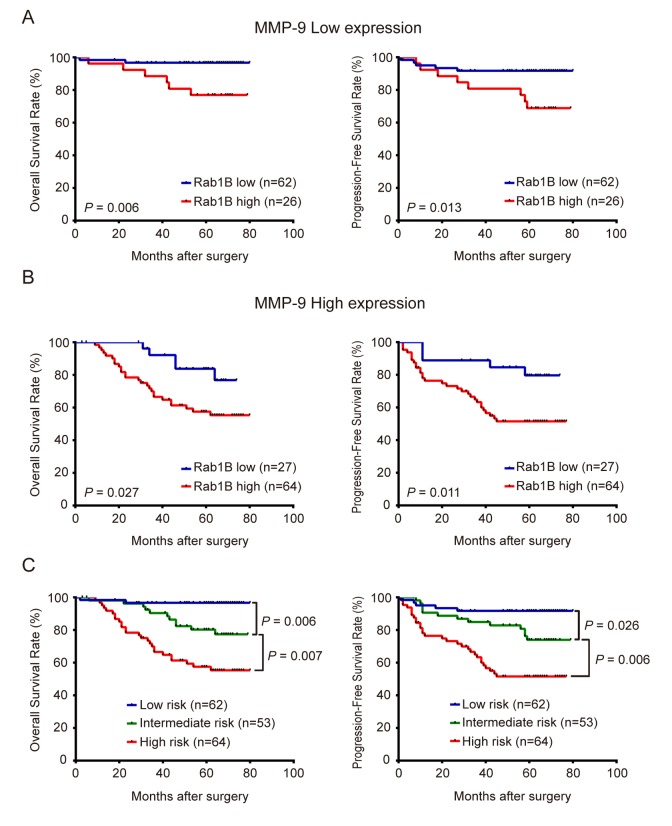
Combined overexpression of Rab1B/MMP9 further improves predictive efficiency for outcome of CRC patients (**A**) OS and PFS of patients with high or low Rab1B expression in those with low-expression of MMP9. (**B**) OS and PFS of patients with high or low Rab1B expression in those with high-expression of MMP9. (**C**) OS and PFS of patients who were stratified into three risk groups by the combined risk score of Rab1B and MMP9 proteins. Kaplan-Meier survival was used to predict the outcomes of CRC patients with low, intermediate or high combined risk score.

We found that in patients with low MMP9 expression, those with high Rab1B expression had significantly worse survival than those with low Rab1B expression (Fig. 4A). Similar result was observed in patients with MMP9 high expression (Fig. 4B). These results suggest that Rab1B is a prognostic predictor independent of MMP9. Therefore, we hypothesize that combination of both proteins will improve their predictive efficiency for survival. To this end, a new combined risk score was calculated as the sum of Rab1B score (0 or 1) and MMP9 score (0 or 1) for each case. Survival analysis shows that patients with low- (score 0), intermediate- (score 1) or high-risk (score 2) have significantly different 5-year overall survival rates, 96.7 %, 80.2 % and 57.5 %, respectively. Multivariate Cox regression analysis reveals that the new combined risk score is an independent prognostic factor for OS and PFS in CRC patients (Table S1). Compared with TNM staging system, Rab1B/MMP9 combined risk score has some better predictive accuracy for OS (ROC area: 0.76 vs. 0.71, Fig. S1A) and PFS (ROC area: 0.73 vs. 0.69, Fig. S1B) of CRC patients though there is no statistically significant difference between the two ROC areas. Thus, the new combined risk score is a potentially useful biomarker that provides additional prognostic information for physician to evaluate survival and make decision on adjuvant chemotherapy in post-operative CRC patients.

### Rab1B and MMP9 protein expression predicts outcome of adjuvant chemotherapy in CRC patients

**Figure 5:**
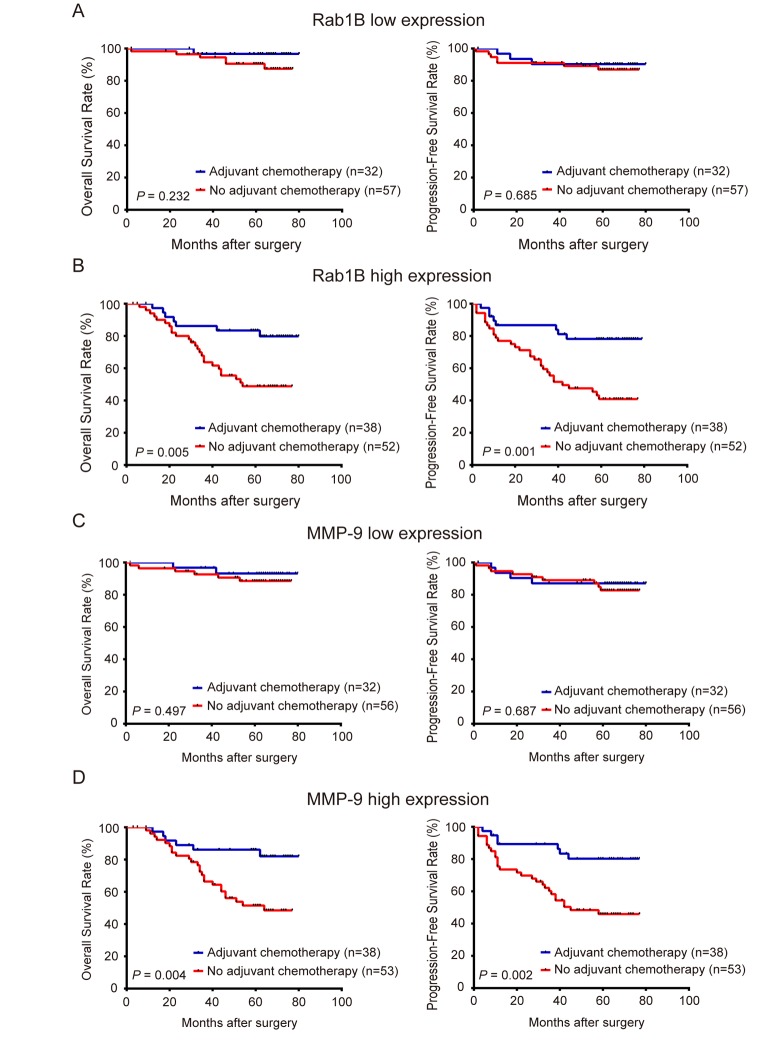
Rab1B and MMP9 protein expression predicts outcome of adjuvant chemotherapy in CRC patients Patients with CRC were stratified into high- or low-expression group by Rab1B or MMP9 expression. (**A**) Kaplan-Meier survival and Log-rank test were used to compare OS and PFS of CRC patients with or without adjuvant chemotherapy in low Rab1B expression group**.** (**B**) OS and PFS of CRC patients with or without adjuvant chemotherapy in the high Rab1B expression group. (**C**) OS and PFS of CRC patients with or without adjuvant chemotherapy in the low MMP9 expression group. (**D**) OS and PFS of patients with or without adjuvant chemotherapy in the high MMP9 expression group.

**Figure 6:**
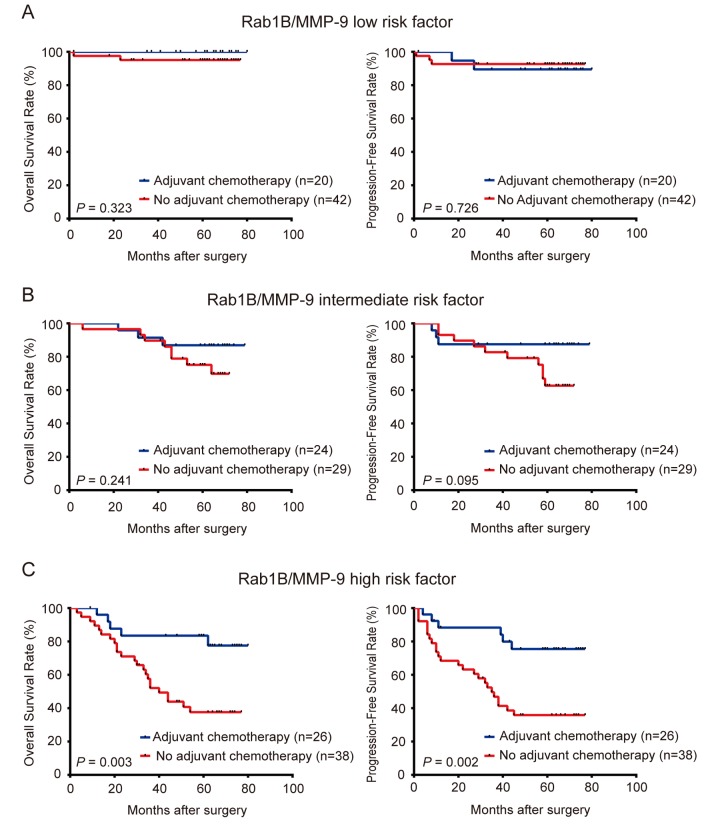
Combined expression of Rab1B and MMP9 proteins predicts the outcome of adjuvant chemotherapy in CRC patients CRC Patients were stratified into three risk groups by the combined risk score of Rab1B and MMP9 protein expression. Kaplan-Meier survival was used to compare OS and PFS of CRC patients with or without adjuvant chemotherapy in low risk group (**A**), intermediate risk group (**B**), and high risk group (**C**).

HR, hazard ratio; CI, confidence interval; Shallow: the depth of tumor invasion within mucosa and muscularis; Deep: tumor invasion to or beyond serosa; TNM, tumour node metastasis; CEA, Carcinoembryonic antigen; CA199, carbo-hydrate antigen 199.

In clinical practice, physicians determine the use of adjuvant chemotherapy based on the clinical stages and high-risk features of patients with CRC. However, clinical stages and high-risk features do not always accurately predict the outcome of chemotherapy. To investigate the utility of Rab1B and MMP9 proteins in CRC management, we explored the use of Rab1B and/or MMP9 protein expression to predict the response to adjuvant chemotherapy in CRC patients. First, we divided patients into high- and low-Rab1B groups. Survival analysis revealed that patients with low Rab1B protein had a similar outcome (including OS and PFS) regardless whether they had adjuvant chemotherapy (Fig. 5A), indicating that these patients do not benefit from adjuvant chemotherapy. Thus these patients should avoid the unnecessary toxicity and economic burden associated with chemotherapy. We next conducted the same analysis on patients with high Rab1B protein. Surprisingly, patients received adjuvant chemotherapy had markedly better OS and PFS than those not received the chemotherapy (Fig. 5B), clearly indicating that patients with high Rab1B protein level may obtain benefit from adjuvant chemotherapy. We also performed the same analysis on MMP9 and observed similar results (Fig. 5C and 5D). Finally, we combined Rab1B and MMP9 into the aforementioned new risk score model and conducted the same analysis. The results show that in patients with low or intermediate risk, there is no difference in OS and PFS between patients with and without chemotherapy (Fig. 6A and 6B), but in patients with high risk, those with chemotherapy had significantly better survival than those without chemotherapy (Fig. 6C).

According to the current criteria of adjuvant chemotherapy, 109 patients were not administered adjuvant chemotherapy in this study. When these patients were divided into low- and high-expression groups based on Rab1B or MMP9 expression, those with high-expression of Rab1B or MMP9 had significantly worse survival (including OS and PSF) than those with low-expression (Fig. S2A and S2B), indicating that patients with high-expression of Rab1B or MMP9 should be administered adjuvant chemo-therapy to improve survival. Next, we combined both proteins to predict outcome of these 109 patients, and the result showed that patients with low combined risk had the best survival and those with high combined risk had the worst survival among the three risk subgroups (Fig. S2C). Finally, we compared single proteins with the combined model using ROC analysis. The result demonstrates that the combination risk score model is remarkably better than single proteins (Fig. S2D), suggesting that the combined risk model can provide more detailed and accurate information to help physicians make chemotherapy decisions.

## DISCUSSION

Rab1A and Rab1B are highly homologous and known to share common biological functions in ER to Golgi trafficking and autophagosome formation [26, 27]. Our previous studies have demonstrated that Rab1A is an oncogene whose overexpression is correlated with poor prognosis in patients with CRC and HCC [10, 11].

Hence, it seems plausible that Rab1B also plays an oncogenic role in CRC. In this study, as expected, we find that the expression of Rab1B mRNA and protein is up-regulated in most of CRC tissues and cell lines, which is consistent with the result recently reported by Zhai et al [17]. Furthermore, another recent study suggested that depletion of Rab1B suppresses tumor growth by inhibiting PI3K/AKT signaling pathway in the lung cancer cell line A549 [28]. However, Rab1B was also reported to be down-regulated in breast cancer and inhibits proliferation and metastasis of breast cancer cells [12]. The reported contradictory functions for Rab1B protein may be due to its distinct roles in a tissue origin- and tumor type-specific manner. Rab1B may participate in distinct signaling pathways, promoting either oncogenic or tumor-suppressing activity in different context.

At present, the most important clinical prognostic predictor of CRC patients is TNM stage. While our data support the clinical utility of the TNM staging system in predicting the prognosis of CRC patients, many CRC patients with same TNM stages have opposite clinical outcomes, suggesting that TNM staging system needs to be improved by additional prognostic factors such as molecular biomarkers for metastasis and relapse. In this study, we explored the prognostic value of Rab1B and MMP9 in CRC patients. Our results demonstrate that Rab1B is a significant prognostic factor independent of the TNM staging system, while MMP9 is a marginally significantly independent predictor for survival of CRC patients, which is consistent with several previous studies [22, 29]. However, when Rab1B and MMP9 are combined into a new risk model, it provides much better prediction of survival in CRC patients. Altogether, our result suggests that the combined risk model is a useful biomarker for prognosis and can provide additional prognostic information in CRC patients.

In clinical practice, TNM staging system also is the main method for making decision on adjuvant chemotherapy. However, our data shows that the patients who are not administered chemotherapy based on the TNM stages have remarkably different survivals when they are stratified by Rab1B or MMP9 expression, indicating that the TNM stage is not adequate for making decision on adjuvant chemo-therapy. In contrast, patients with low Rab1B or MMP9 expression show the similar survival rates no matter whether they received adjuvant chemotherapy or not, suggesting that these patients do not need chemotherapy. In patients with high Rab1B or MMP9 expression, those received chemotherapy have significantly better survival than those did not, indicating that patients with high expression of Rab1B or MMP9 should receive chemotherapy. Furthermore, when combination of Rab1B and MMP9 expression is used to predict survival of patients who did not receive adjuvant chemotherapy based on TNM stage, the combined risk model performs significantly better than Rab1B or MMP9 protein alone. Therefore, our study demonstrates for the first time that Rab1B and MMP9 individually or in combination are useful biomarkers for making decision on adjuvant chemotherapy.

In this study, we reveal that there is a significantly positive correlation between Rab1B and MMP9 expressions in CRC tissues, suggesting that Rab1B and MMP9 have an interaction. Interestingly, it was reported that MMP9 secretion is controlled by Rab1A mediated membrane trafficking [24], suggesting that Rab1B has a similar role on MMP9 secretion. Another recent study reported that PITPNC1 drives metastasis of multiple prevalent cancer types by enhancing Rab1B-mediated vesicular secretion of several pro-metastatic genes, which include MMP1 [30]. It is tempting to speculate that Rab1B regulates MMP9 in such fashion, which warrants further investigation.

In conclusion, our study reveals that the elevated expression of Rab1B and MMP9 is common event in CRC tissues and cell lines. Overexpression of Rab1B and MMP9 alone or together is significantly associated with poor prognosis, suggesting that they are useful biomarkers for prediction of outcome in CRC patients. More importantly, our study demonstrates for the first time that they can accurately predict outcome of adjuvant chemotherapy, which is valuable in guiding the chemotherapy for the post-operative CRC patients. Further studies are required to decipher the molecular mechanism by which Rab1B interacts with MMP9 to promote tumor progression and metastasis in CRC.

## MATERIALS AND METHODS

### Patients and tissue samples

The tumor and matched colorectal tissues were obtained from 179 consecutive patients with stage I-III CRC who underwent radical resection at Sun Yat-Sen University Cancer Center (Guangzhou, China) between January 2009 and December 2010. None of patients received preoperative chemotherapy and/or radiotherapy. After radical resection, patients with stage III and high-risk stage II CRC further received postoperative adjuvant chemotherapy. Fluorouracil-containing regimens are standard, including FOLFOX, XELOX and Xeloda. All the samples were pathologically diagnosed by two experienced pathologists. Histological classification and tumor differentiation were determined according to the criteria of the World Health Organization. Clinical stage was defined according to the 7^th^ edition of tumor-node metastasis (TNM) classification of the American Joint Committee on Cancer Staging (AJCC) [31]. This cohort of CRC patients included 95 males (52.8%) and 85 females (47.2%), with a mean age of 59.5 years old. During the follow-up, tumor assessment was done by colonoscopy, abdominal ultrasound or computed tomography scanning at 3-month intervals for the first 2 years, then at 6-month intervals for 3 years for a total of 5 years. The average follow-up time was 54.2 months, ranged from 1 to 80 months. Overall survival (OS) is defined as the time from the date of surgery to the date of death from any cause or last date of follow-up; Progression-free survival (PFS) is defined as the time from the date of surgery to the date of relapse or metastasis of CRC or death from CRC or last date of follow-up.

Another randomly selected eight pairs of fresh CRC tissues and matched adjacent non-tumorous colorectal tissues from patients undergoing surgical resection in 2011 were collected for immunoblot analysis. This study was reviewed and approved by the Ethical Committees of Sun Yat-Sen University Cancer Center. Written informed consent was obtained from all patients before surgery.

### Cell lines and cell culture

Eleven human CRC cell lines (SW480, SW620, HT29, CACO2, HCT8, HCT116, CW2, DLD-1, RKO, LS174T and LoVo) and human immortalized colon cell lines (CCD-112CoN) were obtained from the American Type Culture Collection (ATCC). All cell lines were cultured in the conditions specified by the manufacturer, and authenticated by short tandem repeat DNA fingerprinting and tested for mycoplasma before use at Medicine Laboratory of the Department of Forensic Medicine, Sun Yat-Sen University (Guangzhou, China).

### Immunohistochemistry (IHC)

IHC staining was performed using standard streptavidin-peroxidase complex method as described previously [32]. Briefly, fresh surgical tissue specimens were fixed in 10% formaldehyde and routinely processed for paraffin embedding. Then these blocks were cut into 4μm thick sections. Endogenous peroxi-dase activity was blocked by 3% hydrogen peroxide in methanol. After antigen retrieval, the sections were incubated with rabbit polyclonal Rab1B antibody (1:200, 17824-1-AP,Proteintech Group, USA) or mouse monoclonal MMP9 antibody (1:50, sc-21733,Santa Cruz, USA) overnight at 4°C and a negative control was set up by replacing the primary antibody with phosphate buffered solution (PBS). Subsequently, the sections were incubated with horseradish peroxidase (HRP)-conjugated anti-rabbit or anti-mouse IgG secondary antibody (Dako, Denmark) for 30 min at room temperature, followed by developing using 3, 5-diaminobenzidine (DAB, Dako, Denmark) substrate and counterstaining with Mayer's hematoxylin for the nuclei.

### Scoring of immunostaining

The scoring of immunostaining was independently performed by two experienced pathologists who were blinded to the clinical information. The immunostaining intensity was scored as 0 - 3 (0, no staining; 1, weak staining; 2, moderate staining; 3, strong staining), and percentage of immunostained tumor cells was scored as 0 - 100. The immunostaining intensity was multiplied by the percentage of stained tumor cells, which resulted in a semi-quantitative immunostaining score (ISS) between 0 and 300.

### Immunoblotting

The fresh CRC tissue samples and CRC cell lines were directly homogenized in RIPA Lysis buffer. 30μg of lysate protein were run on a 10% SDS-PAGE gel and then transferred onto polyvinylidene fluoridemembranes (PVDF, Millipore, USA). After blocking nonspecific binding site with TBST containing 5% non-fat milk, the membranes were incubated with a rabbit polyclonal anit-Rab1B antibody (1:300, sc-599, Santa Cruz, USA), a mouse monoclonal anti-MMP9 antibody (1:200, sc-21733, Santa Cruz, USA) or GAPDH antibody (1:6000, #5174, Cell Signaling Technology, USA) at 4°C overnight. Then the membranes were incubated with HRP-conjugated secondary antibody (1:5000, Jackson Immunoresearch Inc, USA) for about 60 min at room temperature. The blots were scanned and the intensities of protein bands were quantitated by the Bio-Rad software Quantity One (Bio-Rad Laboratories Inc., USA).

### Isolation of total RNA and Real-time quantitative PCR (RT-PCR)

Total RNA from cancer cells was isolated using TRIzol reagent (Invitrogen) as per the manufacturer's instruction. RNA samples were purified and extracted with phenol and chloroform. The quantity of RNA samples was measured by a NanoDrop™ 2000 spectrophotometer (ThermoFisher Scientific, Waltham, MA, USA). One microgram of total RNA was used to synthesize cDNA via reverse transcription reaction according to the protocol of GoScript^TM^ Reverse Transcription System kit (Promega, A5001, Madison, WI, USA). After 1:20 dilution, 2μl of cDNA products was employed for PCR, which was performed on a Roche Lightcycler 96 real time PCR machine (Roche Diagnostics, Indianapolis, IN, USA) according to a standard method as described previously [33]. All samples were amplified in triplicate and GAPDH was detected as an internal control. The relative quantification of target genes was calculated using the comparative 2^-ΔΔCT^ method [34]. Primer sequences for this experiment are as follows:Rab1B forward primer: 5′-GGACTTCAAGATCCGAACCAT-3′, reverse primer: 5′-ATACACCACGATGATGCCA-3′, and the amplicon length: 135bp; MMP9 forward primer: 5′-GGGACGCAGACATCGTCATC-3′, reverse primer: 5′-TCGTCATCGTCGAAATGGGC-3′, and the amplicon length: 139bp; GAPDH forward primer: 5′-CTCCTCCTGTTCGACAGTCAGC-3′, reverse primer: 5′-CCCAATACGACCAAATCCGTT-3′, and the amplicon length: 204bp.

### Statistical analysis

SPSS 17.0 for windows (SPSS, Inc., Chicago, IL, USA) and GraphPad PrismV6 (GraphPad Prism, Inc., USA) were used for statistical analyses. The results were expressed as mean ± SD or SEM. The correlation between Rab1B expression and clinicopathological parameters was analyzed by Chi-square test or Fisher's exact test. The Student's *t*-test was used for the analysis of comparisons. The relationship between Rab1B and MMP9 expressions was tested by Pearson's correlation analysis. The prognostic variables were evaluated by univariate and multivariate Cox proportional hazards regression model. Kaplan-Meier plots and log-rank test were used to estimate cancer specific survival curves.

## SUPPLEMENTARY MATERIALS FIGURES AND TABLES


