# Supplementary material for: Overexpression of Rab1B and MMP9 predicts poor survival and good response to chemotherapy in patients with colorectal cancer
Source: Aging (Albany NY). 2017 Mar 18;9(3):914–29. doi: 10.18632/aging.101200 (PMC5391239; doi:10.18632/aging.101200)
Supplement: Supplementary file 1 [file aging-09-914-s001.pdf]

SUPPLEMENTARY MATERIAL

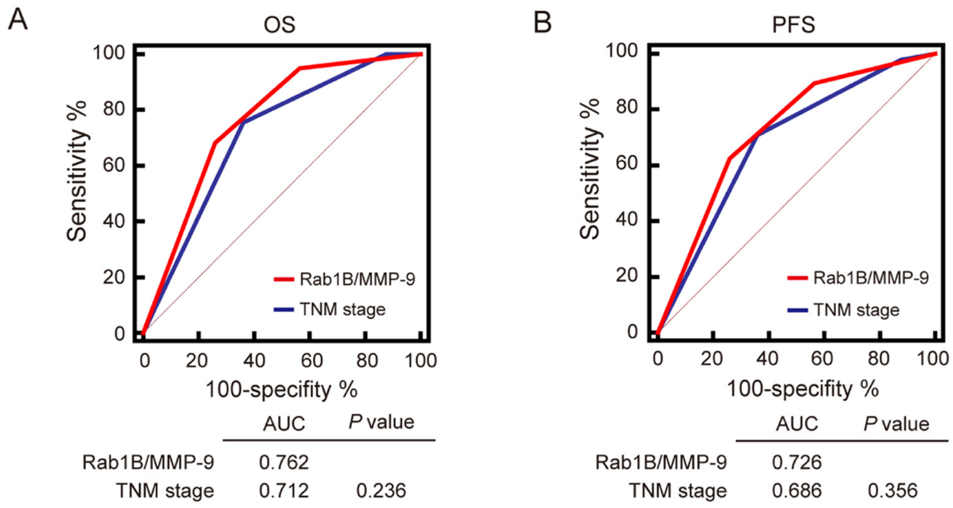

Supplementary Figure 1. Rab1B/MMP9 combined expression has some better predictive accuracy for survival than TNM staging system in CRC patients. Receiver operating characteristic (ROC) curve was used to compare the predictive accuracies for OS (**A**) and PFS (**B**) of CRC patients between combination of Rab1B/MMP9 expression and TNM staging system. AUC, the area under the curve.

Table S1. Univariate and multivariate Cox regression analysis of Rab1B/MMP9 and survival in patients with CRC.

Variables	Univariate analysis		*P* value	Multivariate analysis		*P* value
	HR	95% CI		HR	95% CI	
**Overall Survival**						
Tumor depth (Deep vs. Shallow)	6.477	0.889-47.176	0.065	1.839	0.236-14.320	0.561
TNM stage (III vs. II vs. I)	3.971	2.028-7.778	0.000*	2.217	1.074-4.578	0.031*
Adjuvant chemotherapy (Yes vs. No)	0.375	0.172-0.815	0.013*	0.369	0.166-0.818	0.014*
Rab1B/MMP9 risk factor (Low vs. Intermediate vs. High)	3.272	1.985-5.394	0.000*	2.711	1.605-4.577	0.000*
**Progression-Free Survival**						
Tumor depth (Deep vs. Shallow)	3.914	0.950-16.125	0.059	1.419	0.319-6.324	0.646
TNM stage (III vs. II vs. I)	3.083	1.762-5.394	0.000*	1.852	1.006-3.410	0.048*
Adjuvant chemotherapy (Yes vs. No)	0.428	0.218-0.839	0.013*	0.406	0.203-0.812	0.011*
Rab1B/MMP9 risk factor (Low vs. Intermediate vs. High)	2.604	1.726-3.928	0.000*	2.284	1.473-3.543	0.000*

Shallow: tumor invasion within mucosa and muscularis; Deep: tumor invasion or to beyond serosa; TNM, tumour node metastasis; CRC, colorectal cancer; HR, hazard ratio; CI, confidence interval. *P< 0.05 was considered significant.

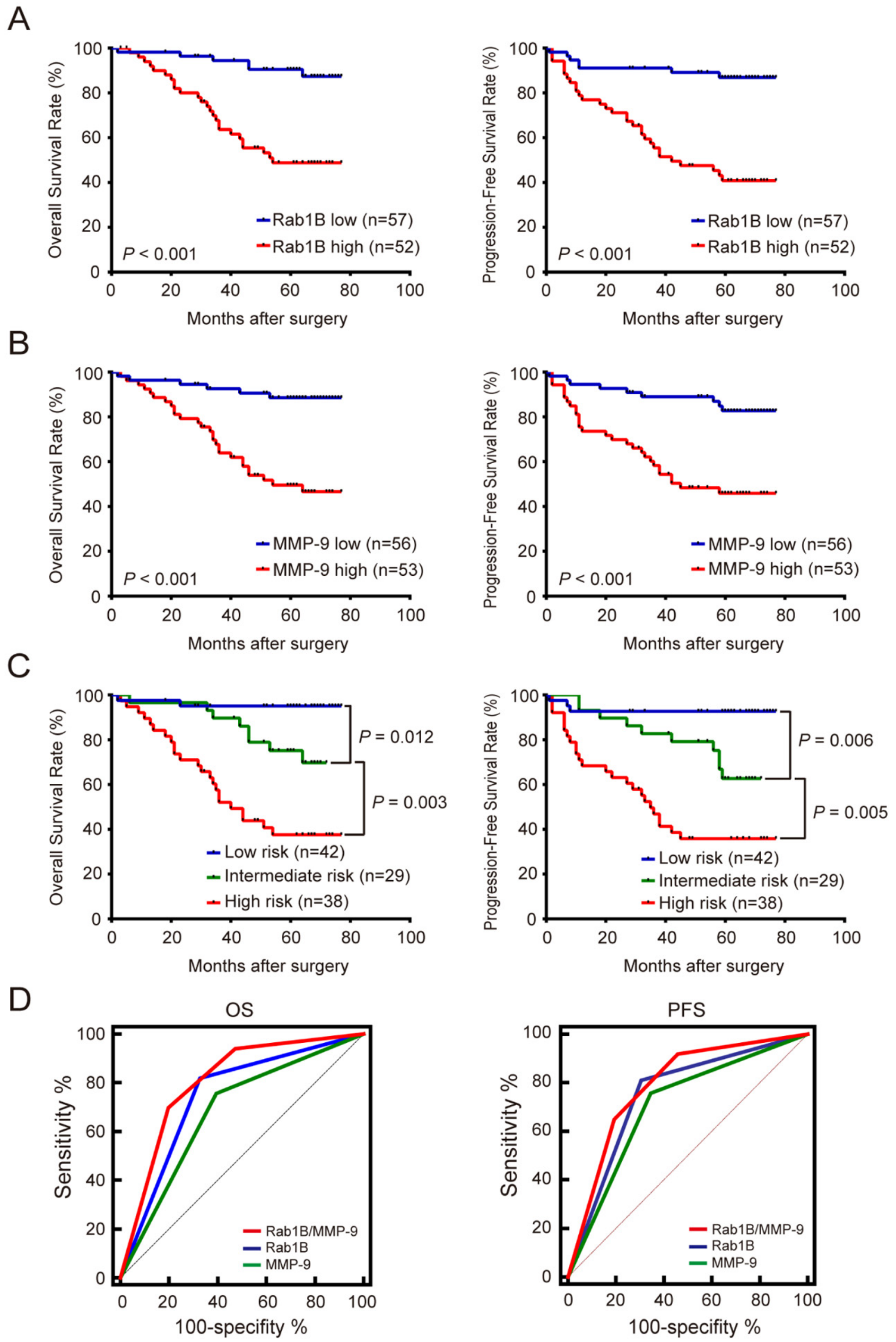

	AUC	*P* value
Rab1B/MMP-9	0.805	
Rab1B	0.745	0.034
MMP-9	0.681	0.002

	AUC	*P* value
Rab1B/MMP-9	0.790	
Rab1B	0.753	0.181
MMP-9	0.705	0.004

Supplementary Figure 2. Either or combination of Rab1B and MMP9 protein expression predicts outcome of chemotherapy in CRC patients. (A) Kaplan-Meier survival analysis shows OS and PFS of CRC patients with high- or low-expression of Rab1B in those without adjuvant chemotherapy. **(B)** OS and PFS of CRC patients with high- or low-expression of MMP9 in those without adjuvant chemotherapy. **(C)** OS and PFS of CRC patients with low-, intermediate- or high-risk score (calculated from the combination of Rab1B and MMP9) in those without adjuvant chemotherapy. **(D)** ROC curve was used to compare Rab1B, MMP9 and the combination of Rab1B/MMP9 in prediction of OS and PFS in CRC patients without adjuvant chemotherapy.
